# Uncertainty quantification for basin-scale geothermal conduction models

**DOI:** 10.1038/s41598-022-08017-2

**Published:** 2022-03-10

**Authors:** Denise Degen, Karen Veroy, Florian Wellmann

**Affiliations:** 1 Computational Geoscience and Reservoir Engineering (CGRE), RWTH Aachen University, Wüllnerstraße 2, 52062 Aachen, Germany; 2 Centre for Analysis, Scientific Computing and Applications, Department of Mathematics and Computer Science, Eindhoven University of Technology (TU/e), Groene Loper 5, Eindhoven, The Netherlands; 3 Faculty of Civil Engineering, RWTH Aachen University, Schinkelstraße 2, Aachen, Germany

**Keywords:** Solid Earth sciences, Geophysics

## Abstract

Geothermal energy plays an important role in the energy transition by providing a renewable energy source with a low CO_2_ footprint. For this reason, this paper uses state-of-the-art simulations for geothermal applications, enabling predictions for a responsible usage of this earth’s resource. Especially in complex simulations, it is still common practice to provide a single deterministic outcome although it is widely recognized that the characterization of the subsurface is associated with partly high uncertainties. Therefore, often a probabilistic approach would be preferable, as a way to quantify and communicate uncertainties, but is infeasible due to long simulation times. We present here a method to generate full state predictions based on a reduced basis method that significantly reduces simulation time, thus enabling studies that require a large number of simulations, such as probabilistic simulations and inverse approaches. We implemented this approach in an existing simulation framework and showcase the application in a geothermal study, where we generate 2D and 3D predictive uncertainty maps. These maps allow a detailed model insight, identifying regions with both high temperatures and low uncertainties. Due to the flexible implementation, the methods are transferable to other geophysical simulations, where both the state and the uncertainty are important.

## Introduction

Geophysical and geoscientific applications have many sources of uncertainties, arising from, for instance, unresolved and unaccounted physical processes, inaccurate geometrical information, and variations in the parameter distributions^1–7^. Identifying and quantifying these uncertainties is a non-trivial process. Methods that easily require a million forward simulations, as Markov chain Monte Carlo (MCMC), make this task not only non-trivial but computationally prohibitive for basin-scale geological heat flow models using state-of-the-art finite element (FE) solvers.

A common way to address this is to replace the finite element model by a surrogate model such as Kriging^8,9^, or polynomial chaos expansions^10^. The issue with these surrogate models is that they are based on observations and do not preserve the physics. Values outside the observation space need to be determined via inter- and extrapolation. For geothermal studies, however, we are interested in the entire temperature distribution at a particular target depth and at preserving the physics to compensate for data sparsity. Therefore, we use a physics-based learning approach, the reduced basis method (RB)^11–14^, as the surrogate model. In contrast to other surrogate models, the RB method has the advantage that it retrieves the temperature distribution in the whole model and thus preserves the physics, enabling an evaluation of the uncertainties in the complete model. Furthermore, the RB method provides, for the here presented geothermal application, an error bound allowing an objective assessment of the approximation quality. This has also advantages in the area of risk assessments since in contrast to data-driven approaches, we are able to provide the accuracy of our model^15^.

The utility of model order reduction for Bayesian inversion has been investigated in previous studies. This includes a data-driven POD approach^16^ and parameter-state model reductions, with a Greedy algorithm, for addressing the computational challenges of uncertainty quantification^17,18^. Furthermore, a POD approach is available for addressing non-linear PDEs^19^. Also, combinations of RB models, to address the computational issues, and error models are discussed^20^. Furthermore, a sparse-grid reduced basis version for Bayesian inversion for both linear and non-linear PDEs exists^21,22^. Additionally, an example of using the RB method within a MCMC scheme for a geodynamical model is available^23^. However, these papers focus on the methodology, and the presented case studies do not capture the typical geometrical complexity of geothermal basin-scale applications.

**Figure 1:**
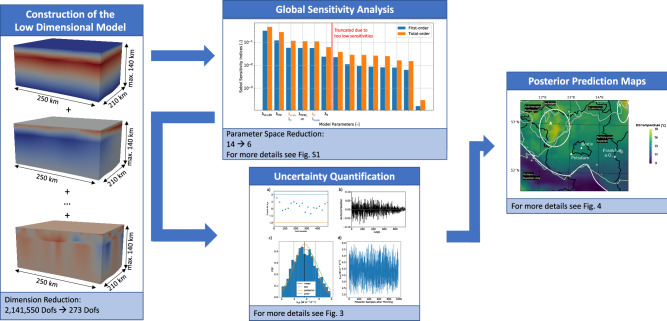
Schematic representation of the workflow. In the left panel, we show exemplarily the first, second, and last basis function of the surrogate (low dimensional) model. The top middle panel shows the results of the global sensitivity analysis (see also Figure  S1), and the middle base panel shows the posterior analysis (see also Fig. 3). The right panel contains the posterior predictive map of the standard deviations highlighting three distinct model areas (see also Fig. 4).

A work investigating the uncertainty of the thermal conductivity via Markov chain Monte Carlo in a geoscientific context is also at hand^24^. Still, in this work, the uncertainty for the temperatures are only considered for five realizations and only interpreted on a 2D-slice lacking the mathematical complexity of uncertainty quantification. In contrast, we present a global-sensitivity-driven stochastic model calibration for complex basin-scale applications to generate predictive 3D uncertainty maps enhancing the efficiency of geothermal exploration. Furthermore, we consider all realizations obtained by the Markov chain Monte Carlo analysis for the uncertainty quantification of the temperatures. The workflow is illustrated in Fig. 1. In previous studies, we investigated the construction of surrogate models for a geoscientific context using the RB method^25^. Furthermore, we demonstrated the benefits of the RB method for basin-scale global sensitivity analysis and deterministic model calibrations^26^.

In this study, we focus on the methodology of uncertainty quantification for geophysical problems. The case study of Berlin–Brandenburg serves as a proof of concept and should highlight the impacts of this methodology for geophysical applications. Although, we focus on a thermal case study, the methods can be applied to a wide range of applications.

The paper is structured as follows: first, we illustrate the methodology and the case study of Berlin–Brandenburg. Afterwards, we present the results of the uncertainty quantification and the predictive uncertainty quantification maps. This is followed by a discussion and concluded afterwards.

## Methods

In the following section, we briefly introduce the numerical methods, the governing equations, and the geological model used throughout this paper.

### Uncertainty quantification

Bayes Theorem is the basis of the Markov chain Monte Carlo (MCMC) method^27^:

1 $$\begin{aligned} P(u | y) \propto P(y | u) \ P(u). \end{aligned}$$

The prior *P*(*u*) describes our knowledge about the unknown value of a parameter without taking the data into account. The posterior *P*(*u*|*y*) is the knowledge we have about the value of *u* given data *y*. Furthermore, *P*(*y*|*u*) is the likelihood, which describes the likelihood of the parameters given the observation data. Often, we do not have a very accurate or detailed knowledge of our unknowns, which means that determining the priors is challenging. MCMC is a method to draw samples from the posterior probability distribution. This is based on the generation of a Markov chain. A Markov chain develops based only on the knowledge of the present and previous events and subsequently iterates to the approximate the posterior distribution. However, this approximations comes at a cost: it often requires thousands to millions of iterations and therefore solves of the forward model^27^.

### The Berlin–Brandenburg model

**Figure 2:**
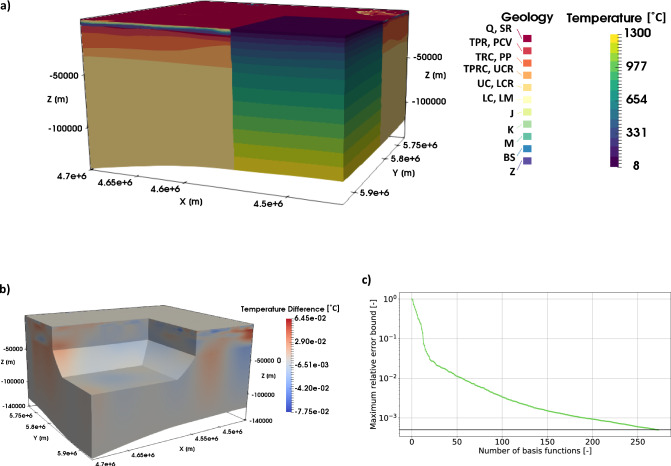
(**a**) Image of the Berlin–Brandenburg model with a partial insert showing the prior temperature distribution. For the layer IDs refer to Table S1. (**b**) The error between the full and reduced model for the prior parameters. (**c**) Convergence of the maximum relative error bound for the entire parameter range.

In this paper, we are using a combination of the Berlin–Brandenburg models presented in two previous studies^28,29^. The model (see Fig. 2) has a spatial extent of 250 km in the EW-direction, 210 km in the NS-direction and extends vertically to the lithosphere-asthenosphere boundary (LAB). It consists of 17 geological layers and is discretized using tetrahedrons. The upper 11 layers have a horizontal resolution of 0.22 km$^{2}$ and a vertical resolution that is interpolated from the *z*-evaluations of the geological layers. The lower six layers have the same horizontal resolution as the upper 11 layers but the vertical element length corresponds to the layer thickness. This results in a tetrahedron mesh with 2,141,550 degrees of freedom.

For the forward simulations, we take a geothermal conduction problem with the radiogenic heat production *S* as the source term^30^:

2 $$\begin{aligned} \lambda \nabla ^2 T + S = 0, \end{aligned}$$

where $\lambda$ is the thermal conductivity, and *T* the temperature. In order to investigate the relative importance of the parameters, and for efficiency reasons, we nondimensionalize the equation, which leads to Eq. (3):

3 $$\begin{aligned} \frac{\lambda }{\lambda _{\text {ref}} \ S_{\text {ref}}} \ \frac{\nabla ^2}{l^2_{\text {ref}}} \ \Bigl ( \frac{T-T_{\text {ref}}}{T_\text {ref}} \Bigr ) \ + \ \frac{S}{S_\text {ref} \ T_{\text {ref}} \ \lambda _{\text {ref}}} \ = \ 0 \end{aligned}$$

Here we chose the maximum thermal conductivity of the Brandenburg model of 3.95 W m$^{-1}$ K$^{-1}$ as reference thermal conductivity $\lambda _\text {ref}$. The maximum temperature of 1300 °C is the reference temperature $T_\text {ref}$, the maximum radiogenic heat production (2.5 $\upmu$W m$^{3}$) is the reference radiogenic heat production $S_\text {ref}$. The reference length $l_\text {ref}$ corresponds to the maximum *x*-extent of all models (250,000 m). At the top of the model, we apply a Dirichlet boundary condition of 8 °C, corresponding to the average annual temperature, and at the base of the LAB a Dirichlet boundary condition of 1300 °C^31^. Additionally, we allow a scaling of the lower boundary condition of ± 10 % to account for errors in the geometric description of the LAB. Note that the LAB has been constrained by using deep seismological studies and by 3D gravity modeling^28^. All thermal properties are summarized in Table S1 and the weak form of Eq. (3) is presented in the next section.

For the validation of the models, we are using the bottom-hole temperature measurements presented in Noack et al.^28,29^ and based on Förster^32^. For the correction, the exact solution to the Bullard line equation has been calculated. Meaning that an exponential integral method has been used, which is based on the modeled temperature build-up during the shut-in time of the wells^32^. The values for the thermal conductivity and the radiogenic heat production are taken from Noack et al.^28,29^ and are originating from previous model studies after Bayer et al.^30^. Throughout this paper, we vary only the thermal conductivities, whereas the radiogenic heat production values are kept constant since the radiogenic heat productions have a minor effect on the temperature distribution at the target depth in comparison to the thermal conductivities. We further reduce the number of involved parameters in the reduction and inverse processes by combining layers with equal thermal conductivities into one, as presented in Table S1. This reduction is necessary to compensate for the small amount of measurement data, which is insufficient to treat all parameters individually. Therefore, all analyses return combined effects of these layers.

### Berlin–Brandenburg—reduced model

We construct a surrogate model using the RB method based on the full FE model with an accuracy of 5 $\times$ 10$^{-4}$ (see Fig. 2c). The RB method is a model order reduction technique that aims at significantly reducing the spatial and temporal degrees of freedom of, for instance, finite element problems. For further information regarding the method please refer to the literature^11–14^, and for more information on the RB method in the context of Geosciences refer to Degen et al.^25^. The geothermal problem, described in Eq. (2), is affine decomposable, meaning separable into a parameter-independent and -dependent part.

The RB method takes advantage of this affine decomposition in an offline-online procedure. During the offline stage, performed only once, all expensive pre-computations for the basis construction are performed. The construction of the basis is achieved via a greedy algorithm^12^, which involves training or “learning” of the low-dimensional model. In contrast to machine learning approaches, we are not training based only on data but instead also consider the physical model.

On the other hand, the online stage uses only the reduced model. Hence, it is for the given example several orders of magnitude faster than the original FE model making it advantageous for “outer loop” processes, such as calibrations and uncertainty quantification.

We derive the weak formulation, where $u(\mu ) \in X$ satisfies^11,13,14^:

4 $$\begin{aligned} a(u(\mu ),v;\mu )=f(v;\mu ), \qquad \forall v \in X. \end{aligned}$$

Note that we use the operator representation here. This means, we present the bilinear form *a* (instead of the stiffness matrix) and the linear form *f* (instead of the load vector). In particular, the bilinear form *a* has the following decomposition:

5 $$\begin{aligned} a(w,v;\lambda ) = \sum _{q=0}^{n} \lambda _q \int _{\Omega } \nabla w \ \nabla v \ d\Omega , \qquad \forall v,w \in X, \ \forall \lambda \in \mathcal {D}, \end{aligned}$$

where *w* is the trial function, *v* the test function, the index “*q*” denotes the number of the training parameters (for more information see Table S1), *X* the function space ($H_0^1(\Omega ) \subset X \subset H_1(\Omega )$), $\Omega$ the spatial domain in $\mathbb {R}^3$, and *D* the parameter domain in $\mathbb {R}^{p}$ with *p* being the number of parameters. In our example *p* is equal to 14. The linear form *f* is decomposed in the following way:

6 $$\begin{aligned} \begin{aligned} f(v;\lambda , s) =& \sum _{q=0}^{n} \lambda _q \ s \int _{\Gamma } \nabla v \ g(x,y,z) \ d\Gamma +&\ s \int _{\Gamma } \nabla v \ S \ d\Gamma , \qquad \forall v \in X, \ \forall \lambda \in \mathcal {D},\\ \text {with} \ g(x,y,z) =&\ T_\mathrm{{top}} \frac{h(x,y,z)-z_\mathrm{{bottom}}(x,y)}{d(x,y)}. \end{aligned} \end{aligned}$$

Here, $\Gamma$ is the boundary in $\mathbb {R}^{3}$, *s* the scaling parameter for the lower boundary condition, *g*(*x*, *y*, *z*) the lifting function, $T_\mathrm{{top}}$ the temperature at the top of the model, *h*(*x*, *y*, *z*) the location in the model, $z_\mathrm{{bottom}}(x,y)$ the depth of the bottom surface, and *d*(*x*, *y*) the distance between the bottom and top surface.

## Results

For the uncertainty quantification of the Berlin–Brandenburg model, we perform a Markov chain Monte Carlo analysis^27^ with a Metropolis sampling using the Python library PyMC^33^. A previously performed Sobol sensitivity analysis with the Saltelli sampler and 300,000 forward solves showed that the model is insensitive to eight of the 14 parameters (Fig. S1)^34^. We thus reduce the parameter dimension from 14 parameters to six. For more information regarding global sensitivity analyses, refer to Sobo^35^, and Degen et al.^26^.

For all thermal conductivities in the sensitivity analysis and the MCMC algorithm, we allow a variation of ± 50%. The number of function evaluations for the MCMC run is set to 1,000,000 with a thinning of 1000 and 10,000 burn-in-simulations. For the priors, we use normally distributed parameters. The mean of each parameter corresponds to the fitted thermal conductivity values of Noack et al.^28,29^. Both the standard deviation and proposal standard deviation are set to:

- one for the Tertiary-pre-Rupelian-clay/Upper Cretaceous and Lower Cretaceous/Jurassic layer
- two for the Keuper layer
- four for the Zechstein layer and the Lithospheric Mantle
- 0.002 for the scaling parameter of the lower boundary condition

and are afterwards divided by their respective mean values. The standard deviations have been determined such that the values do not exceed a range of ± 50% of their mean values to ensure physical plausibility. For the stochastic model calibration, we use the temperature data presented in Noack et al.^28,29^. The bottom-hole temperatures of this database have been measured during the drilling process and are based on Förster^32^. This correction might not fully capture the perturbation of the temperature field. Therefore, we apply a standard deviation of 2% for the observation data.

### Thermal conductivities

Now, we discuss the posterior distribution of the thermal conductivities obtained by the MCMC analysis (Table S1). Through a Quantile–Quantile analysis (Fig. S2), we determined that the normal distributions describe our parameter quite well. Hence, we discuss in the following only the posterior mean and standard deviations of the thermal conductivities.

We obtain for the Tertiary Rupelian-clay/Upper Cretaceous layer (Fig. S4), a slight increase in the posterior mean thermal conductivity of 0.05 W m$^{-1}$ K$^{-1}$ in contrast to the prior thermal conductivity. The parameter follows a normal distribution with a standard deviation of 0.47 W m$^{-1}$ K$^{-1}$. We observe a posterior thermal conductivity of:

- 2.11 W m$^{-1}$ K$^{-1}$ ± 0.45 W m$^{-1}$ K$^{-1}$ for the Lower Cretaceous/Jurassic/Buntsandstein layer (Fig. S5),
- 2.35 W m$^{-1}$ K$^{-1}$ ± 0.58 W m$^{-1}$ K$^{-1}$ for the Keuper layer (Fig. S6),
- and 3.56 W m$^{-1}$ K$^{-1}$ ± 0.81 W m$^{-1}$ K$^{-1}$ for the Zechstein layer (Fig. S7).

**Figure 3:**
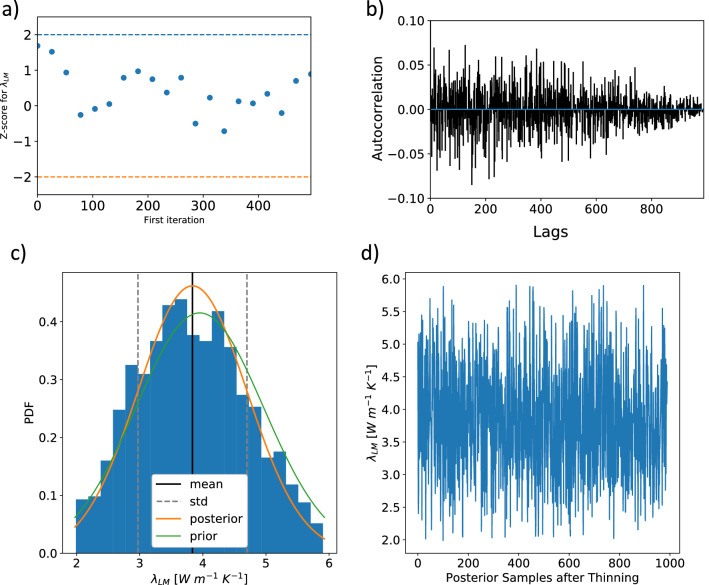
Posterior analysis of the lithospheric mantle (LM) as an example. The remaining posterior analyses figures are found in the Supplementary Material. Shown are the (**a**) Geweke Plot (**b**) autocorrelation, (**c**) posterior parameter distributions, and (**d**) the trace.

Hence, all three cases show an increase in the posterior thermal conductivity in comparison to the prior thermal conductivity, and they are also normally distributed, as visually determined from the quantile-quantile plots in the Supplementary Material. The Lithospheric Mantle shows a decrease in the posterior mean thermal conductivity of 0.11 W m$^{-1}$ K$^{-1}$ in comparison to the prior thermal conductivity and has a posterior standard deviation of 0.86 W m$^{-1}$ K$^{-1}$ (Fig. 3). The scaling parameter (Fig. S8) has a posterior mean value of 1.00, which is identical to the prior value, and a posterior standard deviation of 0.04. All parameters follow a normal distribution and an autocorrelation around zero. The *z*-scores (Fig. 3a, Figs. S4a– S8a) indicated converges for all chains. The *z*-scores measure the mean and the variance of the entire chain.

### Uncertainty quantification maps

First, we use the parameter distributions of the MCMC analysis to generate 2D and 3D uncertainty quantification maps. We make here also use of the RB method, which allows us to compute model realizations for samples from the posterior distribution to obtain temperature state values everywhere in space.

For the generation of uncertainty quantification maps, we have to choose a suitable representation. A Quantile–Quantile analysis (Fig. S3) for nine points at a depth of 5 km shows that the temperature is normally distributed. Hence, we plot the posterior mean temperatures and their standard deviations to achieve a suitable representation of the temperature uncertainties in the following.

**Figure 4:**
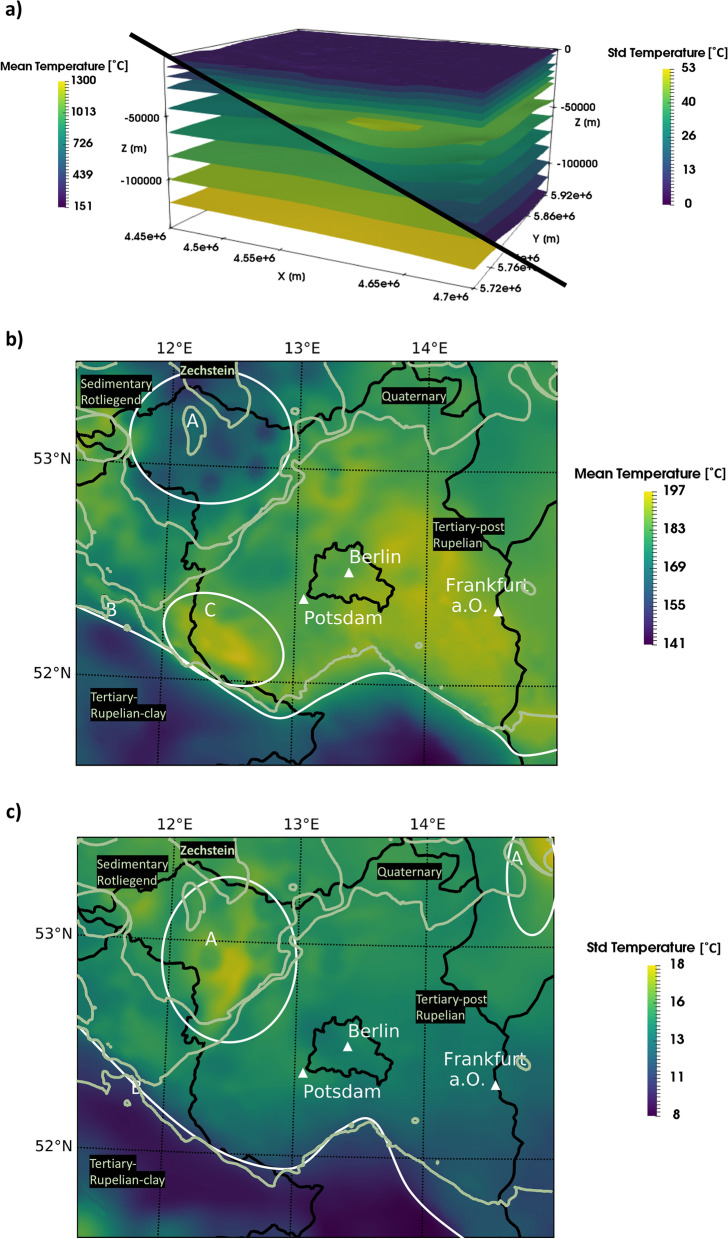
(**a**) Distribution of the posterior mean temperature and the posterior standard deviation of the entire Berlin–Brandenburg model. (**b**) Map of the posterior mean temperature and (**c**) posterior standard deviation at the target depth of 5 km. The light green lines in (**b**,**c**) indicate the boundaries of the geological layers. The maps in (**b**,**c**) have been generated using ParaView^36^ and the Python library BaseMap^37^.

First, we present the posterior distributions in the entire Berlin–Brandenburg model. The posterior standard deviations have their highest value within the sedimentary basin at a depth of about 30–35 km (see Fig. 4a). Consequently, the highest uncertainties also occur there. Overall, we observe uncertainties ranging from 0 to 53 °C. We observe that the uncertainty decreases towards the upper and lower model boundaries and increases towards the center part of the model. For a detailed discussion of this aspect please refer to the “Discussion” section. The gradient of the posterior mean temperature distribution is steep in the upper part of the model and has a significantly less steep gradient in the lower part of the model. The temperatures range from 8 to 1300 °C (see Fig. 2).

Now, we focus on the posterior distributions at a typical target depth for geothermal systems of 5 km. The posterior mean temperature ranges from 141 to 197 °C, and the posterior standard deviation from 8 to 18 °C. The highest uncertainty, in a depth of 5 km, is north of the interface of the Tertiary-post-Rupelian and the Rupelian clay and south to the Zechstein—Sedimentary Rotliegend interface. The area is marked with an A in Fig. 4c. It has its highest peak southeast to the region, where salt structures majorly influence the posterior mean temperatures. Generally, from the interface (marked with a B), the uncertainties increase towards the north and decrease towards the south of the model.

The highest posterior mean temperatures of over 190 °C are north of the interface of the Tertiary-post-Rupelian and the Rupelian clay (marked with a C). In contrast, the lowest posterior mean temperature values around 140 °C are south of this interface (see B in Fig. 4b). In general, the posterior mean temperature north of the interface decrease to the northern border of the model. Furthermore, in the north-west part of Berlin–Brandenburg, a region of lower posterior mean temperatures is located (area A in Fig. 4b). We explain the reasons for this decreased posterior mean temperature in the “Discussion” section.

### Computational cost

The reduction requires 273 basis functions for reaching the pre-defined relative error tolerance of 5 $\times$ 10$^{-4}$ for the nondimensional model (see Fig. 2c). Note that the most accurate measurements have an accuracy of 10$^{-1}$. Consequently, the chosen error tolerance ensures that we do not introduce approximation errors above the measurement error. The reduced basis method leads to a speed-up of 1.0 $\times$ 10$^{5}$. This yields an execution time of the MCMC algorithm of about 4.5 h, for the one million forward solves.

The RB method requires 5.4 h for the offline stage, using two Intel Xeon Platinum 8160 CPUs (24 cores, 2.1 GHz, 192 GB of RAM) and 4.5 h for the MCMC method. Note that with the finite element method itself, the same analysis would require over 16 core-a.

## Discussion

A benefit of the methodology presented here is the generation of predictive uncertainty quantification maps, enabled by using the RB method as a surrogate model. Therefore, we are able to reveal important insights into the spatial distribution of the uncertainties. Most other surrogate models would not allow the generation of predictive physics-preserving uncertainty maps for the entirety of the model since they generally do not preserve the physics.

### Thermal conductivities

To discuss the uncertainties related to the thermal conductivities, we first focus on the posterior mean thermal conductivities. The posterior mean thermal conductivities of all layers show only a slight deviation from the prior thermal conductivities. This is not surprising since they are derived from previous model studies and are therefore already well adapted to the model. However, if we compare them to the measured thermal conductivities presented in Noack et al.^28^, we observe an apparent deviation since this paper present the input parameters prior to the “trial-and-error” model calibration.

Even though the posterior mean thermal conductivities are in a good agreement with the prior thermal conductivities, the need for uncertainty quantification becomes apparent through the posterior standard deviation. For all layers, we observe large posterior standard deviations for the thermal conductivity, meaning that we have high uncertainties for all layers. The uncertainty in the parameters is mainly influenced by the uncertainty of the observation data and by the upper boundary condition. In our study, we place a lot of trust in the data. Still, we allow variations from that data set since we are operating with partially corrected bottom-hole temperatures. We assume that the correction factor is not able to fully compensate for the perturbation of the temperature field during the drilling process, resulting in slightly uncertain observation data. The posterior standard deviation decreases by placing more trust in the observation data. Therefore, temperature observations that are performed when the temperature field is in equilibrium would significantly improve the certainty of the different thermal conductivities.

Except for the Lithospheric Mantle, all posterior mean thermal conductivities show an increase in comparison to the prior thermal conductivity. Since the layers above the salt show an increase in the posterior thermal conductivity and the layer below shows a decrease, that might be an indication that some salt structures were not resolved. The stochastic calibration demonstrates that a geothermal conduction problem adequately describes the sedimentary basin of Berlin–Brandenburg. Furthermore, the small posterior standard deviation of the scaling parameter for the lower boundary condition shows that the boundary is placed far enough from the area of interest to avoid any interference.

### Uncertainty quantification maps

We first focus on the uncertainties associated with the temperatures in the entire Brandenburg model. The distribution of these uncertainties seems to be contradictory to our expectations. Usually, one expects an increasing uncertainty with depth. We observe a decreasing uncertainty towards the boundaries and an increasing uncertainty towards the center part of the model instead. Both for the top and the bottom boundary condition, we apply Dirichlet boundary conditions, where the upper boundary condition has a value of 8 °C throughout all simulations. The lower boundary condition varies by a factor of ± 10%. We allow this variation to account for geometrical parameterization errors of the LAB. This is the reason why we observe decreasing uncertainties towards these boundary conditions because the values of the boundaries are relatively fixed within all simulations.

The highest uncertainties are between 30 and 35 km depth, where no interactions of the boundary conditions are observable. For a detailed investigation of the influence of boundary conditions on a geothermal conduction model, we refer to Degen et al.^34^.

We can also use the distribution of the uncertainties to investigate the influence of the respective boundary conditions. Although the LAB is at a depth varying from approximately 100 to 140 km, the boundary significantly influences the model up to a depth of 80–100 km. For our investigations, this is uncritical since our target depth is at 5 km depth. Nonetheless, this demonstrates that it is essential to have a vertical extent that is significantly larger than the target depth. The upper boundary condition is influencing the model to a depth of 10 km, meaning that the upper boundary condition significantly affects our target depth. This is not avoidable since the surface naturally defines the upper boundary. However, this is less critical than the influence of the lower boundary condition because we can determine the upper boundary with a much higher certainty than the lower. Nonetheless, it shows that it is crucial to characterize the upper boundary condition with great detail. This means that we need to think about changing the type of boundary condition that we use. Therefore, we could, for instance, employ a Robin boundary condition to allow an interaction between the subsurface and the atmosphere. A previous study^38^ has shown that a variation of the value of the boundary condition does not majorly impact the sensitivities of the model response as long as we consider a Dirichlet boundary condition. This is the reason because the value of the boundary condition is the same in every realization.

At the target depth, the highest uncertainties are in the northwest (denoted by “A” in Fig. 4). Hence, they are north of the Tertiary-post-Rupelian and the Rupelian clay interface (denoted by “B” in Fig. 4), and south of the Sedimentary Rotliegend and Zechstein interface. The reason is that the variations of the contrast in thermal conductivity are high at these interfaces. Note that the Rupelian clay has a posterior mean thermal conductivity of 1.93 W m$^{-1}$ K$^{-1}$ with a posterior standard deviation of 0.53 W m$^{-1}$ K$^{-1}$ and the Zechstein layer a posterior thermal conductivity of 3.60 ± 0.96 W m$^{-1}$ K$^{-1}$. Furthermore, the highest uncertainties are adjacent to the region of the salt structures, further emphasizing the influence of the Zechstein layer on the uncertainties. At the target depth, we consider only the Rupelian clay and the Zechstein layer as uncertain and do not include other layers in the uncertainty quantification. The sensitivity analysis shows that the model is insensitive to these parameters. Consequently, the observed uncertainty is arising from the contrast in thermal conductivity between the Rupelian clay- Zechstein layer and the remaining layers.

The posterior mean temperatures at a depth of 5 km are higher north from the Tertiary-post-Rupelian and the Rupelian clay interface (marked with the letter B in Fig. 3b) because the Tertiary-post-Rupelian has a lower thermal conductivity than the Rupelian clay. The colder posterior mean temperature values in the north-western part of the model (area A in Fig. 3b) are coming from the high thermal conductivity of the Zechstein layer. It is further emphasized by the round dome structures in the temperature distribution that are typical for salt. The posterior mean temperature after the stochastic model calibration only slightly deviates from the prior temperature distribution since the changes in the posterior mean thermal conductivity are also minor.

### Reduced order model

The results show that the usage of a physics-based learning approach has considerable advantages for geothermal investigations and similar advantages can be expected for many other geophysical applications. This is caused by the sparsity of the observation data. The data sparsity makes purely data-driven approaches in many geophysical applications prohibitive. Instead of using data for the training phase, we use only the physical model in the construction of the surrogate model and are therefore able to mitigate the problem with the data sparsity at this stage. The data is introduced only during the inversion itself.

## Conclusion and outlook

We presented an uncertainty quantification at the basin-scale with the generation of uncertainty quantification maps. This is computationally possible since we replace the finite element forward simulation by the reduced basis forward simulation. This results in a reduction of computation time from a couple of hundred seconds to a few milliseconds per simulation, and hence in a speed-up of five orders of magnitude. Therefore, we are able to efficiently perform both global sensitivity and MCMC analyses which both require thousands to millions of forward evaluations. Because we consider not only the deterministic but the stochastic temperature distribution, we are able to predict the temperatures with uncertainty, everywhere in space. For future work, it would be interesting to incorporate these temperature uncertainties into the economic evaluation of potential geothermal wells. It would be also interesting to investigate the effects of different observation data qualities on the uncertainty of the model temperature distributions. Another interesting aspect is, to also include structural uncertainties. The methodology as presented here has been already applied to consider geometrical parameters^13,39–41^. The bottleneck of considering structural uncertainties is the mesh generation step, which is very time-consuming. The presented methodology addresses this problem by mapping the difference of every new configuration to a reference mesh. Hence, it deforms the original mesh and avoids a re-meshing step.

## Supplementary Information

Supplementary Information.
