# Supplementary material for: Uncertainty quantification for basin-scale geothermal conduction models
Source: Sci Rep. 2022 Mar 10;12:4246. doi: 10.1038/s41598-022-08017-2 (PMC8913627; doi:10.1038/s41598-022-08017-2)
Supplement: Supplementary file 1 — Supplementary Information. [file 41598_2022_8017_MOESM1_ESM.pdf]

Supporting Information for "Uncertainty Quantification for Basin-Scale Geothermal Conduction Models"

Contents of this file

1. Table S1
2. Figures S1 to S8

INTRODUCTION

This supporting material provides additional information regarding the thermal properties of the Berlin-Brandenburg model in Table S1. In Figure S1, we present the results of the sensitivity analysis, which servers as a preparational study for the uncertainty quantification. Figures S2 and S3 show the quantile-quantile plots for the thermal parameters and the temperatures at designated points in the model, respectively. We present the posterior analysis of the not in the main manuscript presented thermal parameters in Figures S4 to S8 as referenced in the main manuscript.

TABLE S1: THERMAL PROPERTIES

Table S1. Thermal properties of the Berlin-Brandenburg model before and after the uncertainty quantification. The prior thermal properties are from [1, 2]. We denote all parameters that are not involved in the uncertainty quantification, due to too low sensitivities, with n/a. Additionally, the affine decomposition for the model reduction is provided. Note that μ denotes the training parameter, S the radiogenic heat production, λ_{prior} the prior thermal conductivity, λ_{mean} the posterior mean thermal conductivity, and λ_{std} the posterior standard deviation of the thermal conductivity.

ID	Layer	μ	S $[\mu W m^{-3}]$	λ_{prior} $[W m^{-1} K^{-1}]$	λ_{mean} $[W m^{-1} K^{-1}]$	λ_{std} $[W m^{-1} K^{-1}]$
Q	Quaternary	0	0.7	1.50	n/a	n/a
TPR	Tertiary-post-Rupelian	0	0.7	1.50	n/a	n/a
TRC	Tertiary Rupelian-clay	1	0.45	1.00	n/a	n/a
TPRC	Tertiary-pre-Rupelian-clay	2	0.3	1.90	1.95	0.47
UC	Upper Cretaceous	2	0.3	1.90	1.95	0.47
LC	Lower Cretaceous	3	1.4	2.00	2.11	0.45
J	Jurassic	3	1.4	2.00	2.11	0.45
K	Keuper	4	1.4	2.30	2.35	0.58
M	Muschelkalk	5	0.3	1.85	n/a	n/a
BS	Buntsandstein	3	1.0	2.0	2.11	0.45
Z	Zechstein	6	0.09	3.50	3.56	0.81
SR	Sedimentary Rotliegend	7	1.0	2.16	n/a	n/a

PCV	Permo-Carboniferous Volcanics	8	2.0	2.50	n/a	n/a
PP	Pre-permian	9	1.5	2.65	n/a	n/a
UCR	Upper crust	10	2.5	3.10	n/a	n/a
LCR	Lower crust	11	0.8	2.70	n/a	n/a
LM	Lithospheric Mantle	12	0.03	3.95	3.84	0.86
	Parameter	μ	Prior Value [-]		Posterior Mean Value [-]	Posterior Std Value [-]
	Scale	13	1.00		1.00	0.04

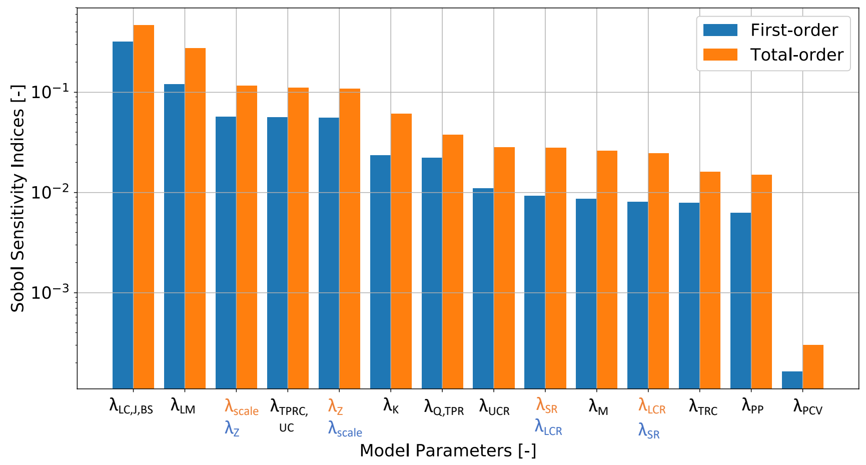

Fig. S1. Global Sensitivity analysis for the Berlin-Brandenburg model. We show the first- (blue) and total-order contributions (orange).

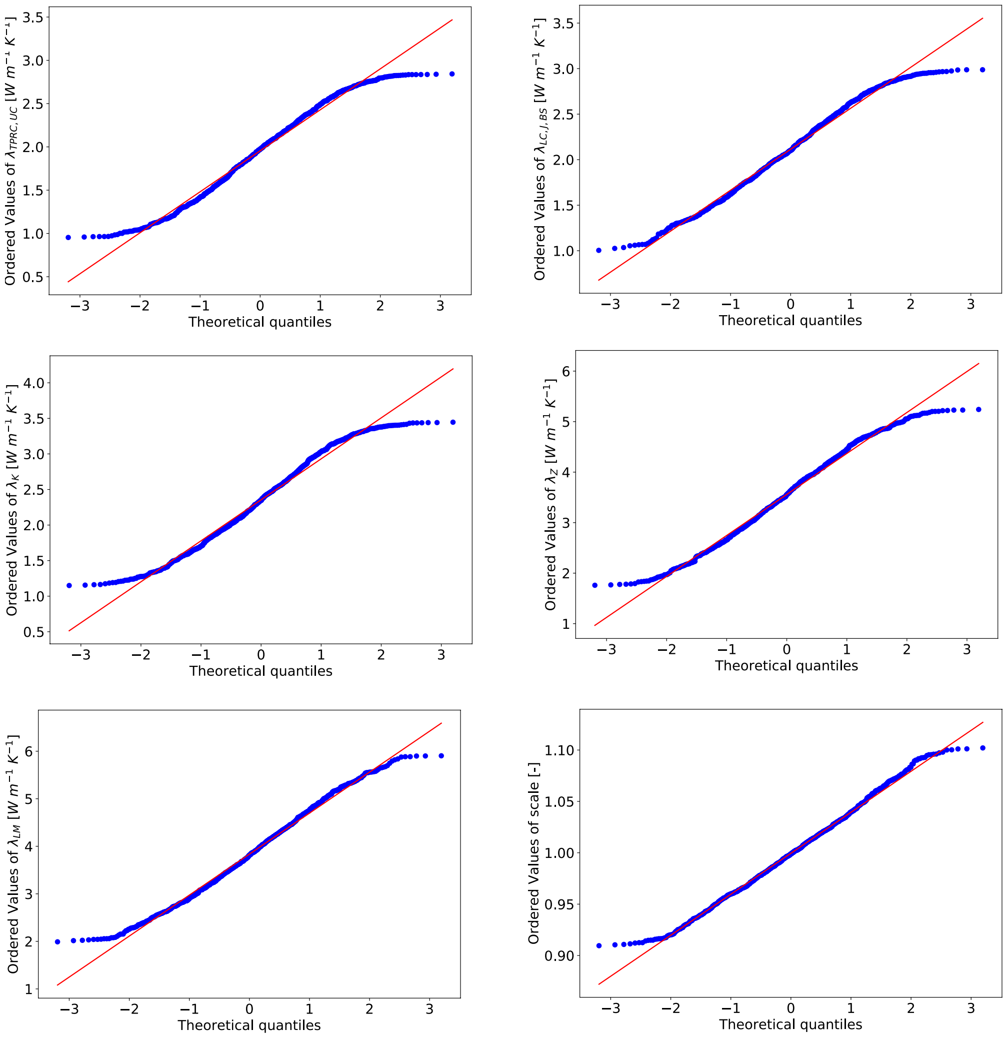

Fig. S2. Quantile-Quantile plots for all thermal conductivities considered in the uncertainty quantification of the Berlin-Brandenburg model.

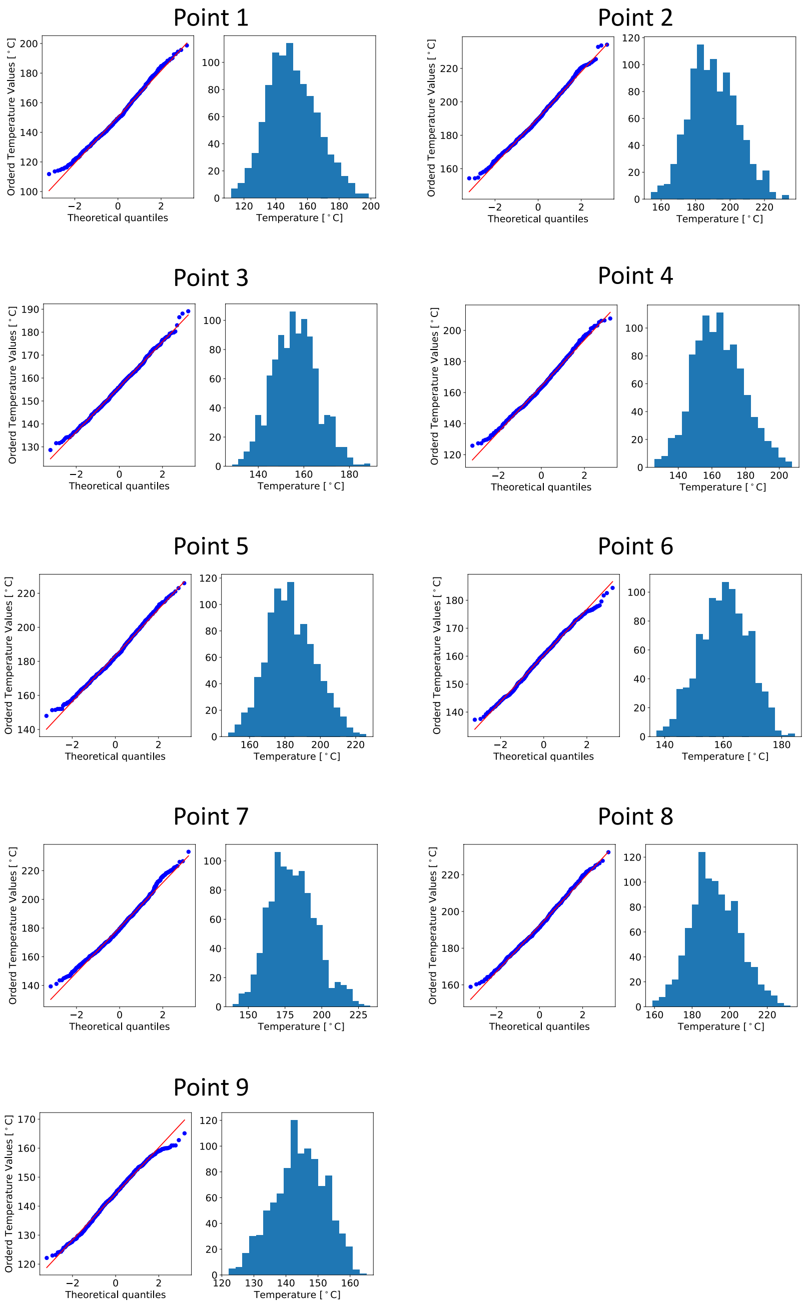

Fig. S3. Quantile-Quantile plots and histograms of the temperatures for all parameters from the MCMC analysis for the Berlin-Brandenburg model at nine points.

$\lambda_{TPRC,UC}$

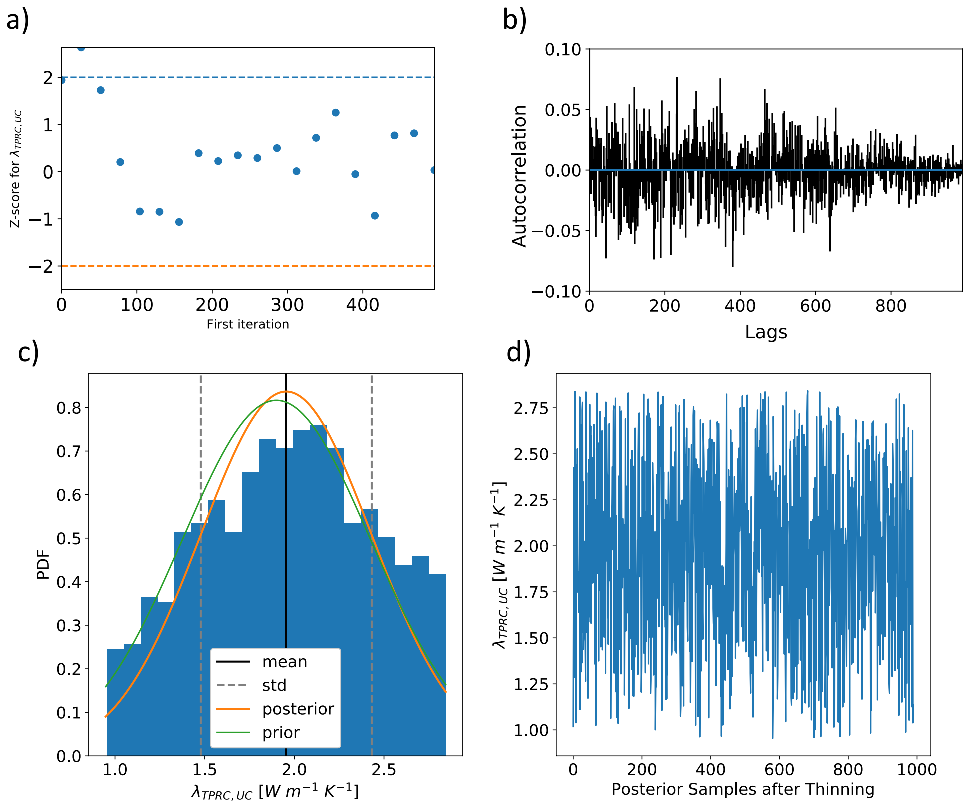

Fig. S4. Posterior Analysis of the Tertiary-pre-Rupelian-clay (TPRC) and the Upper Crust (UC). Shown are the a) Geweke Plot b) autocorrelation, c) posterior parameter distributions, and d) the trace.

$\lambda_{LC,J,BS}$

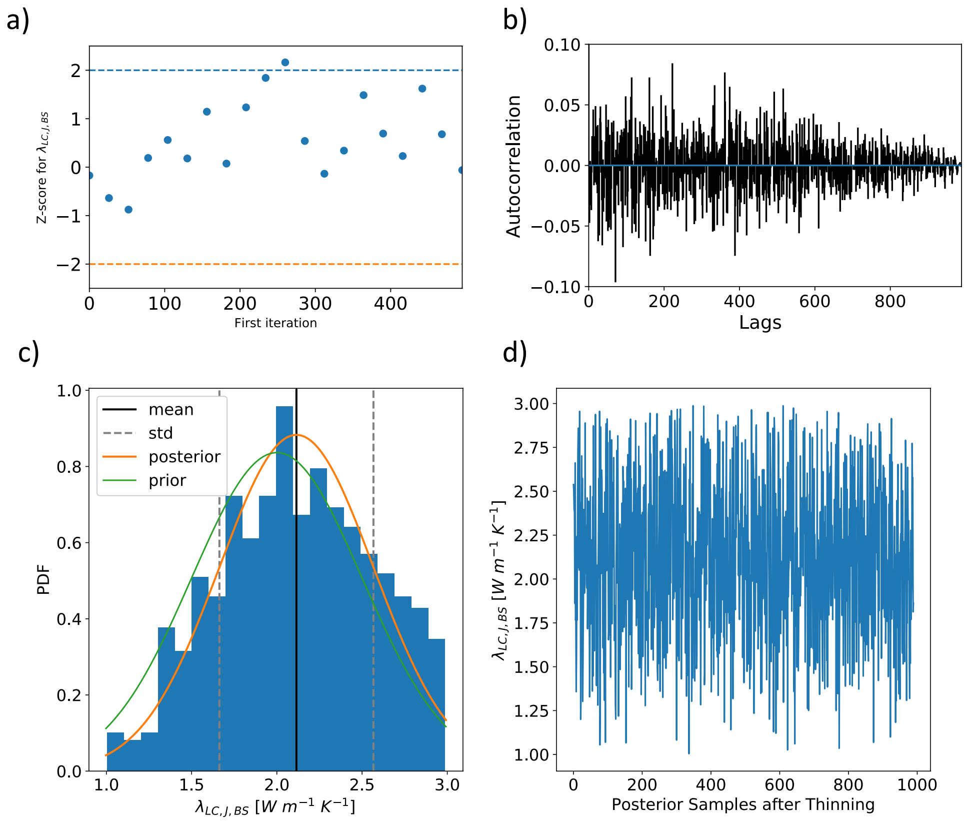

Fig. S5. Posterior Analysis of the Lower Crust (LC), the Jurassic (J), and the Buntsandstein (BS). Shown are the a) Geweke Plot b) autocorrelation, c) posterior parameter distributions, and d) the trace.

λ_K

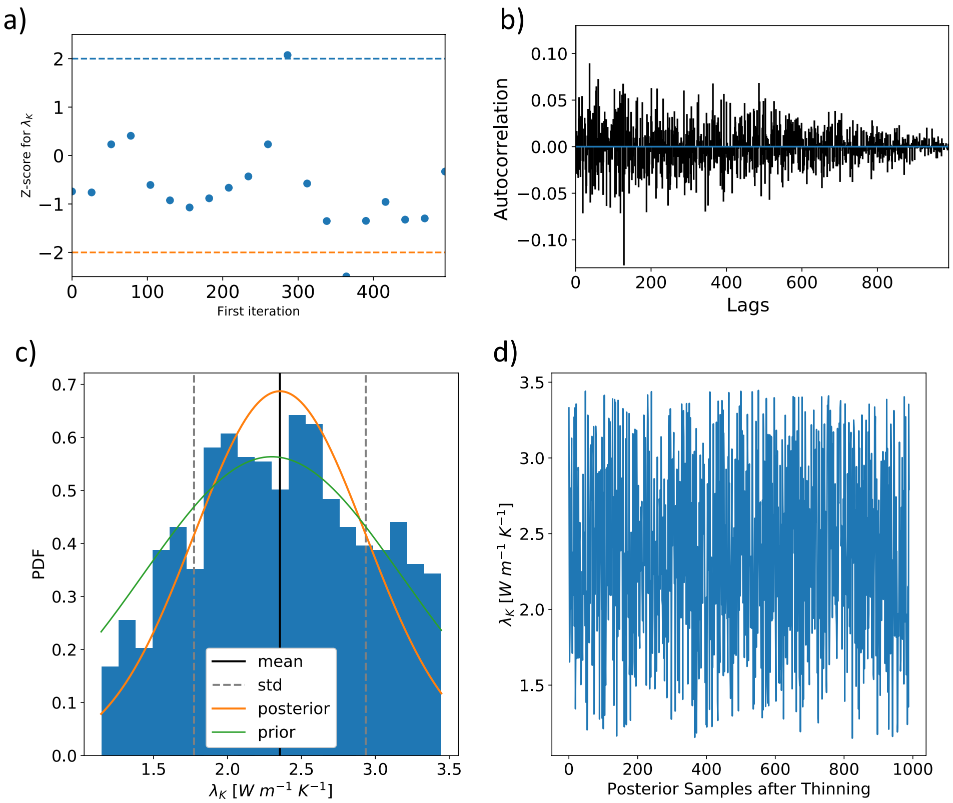

Fig. S6. Posterior Analysis of the Keuper (K). Shown are the a) Geweke Plot b) autocorrelation, c) posterior parameter distributions, and d) the trace.

λ_z

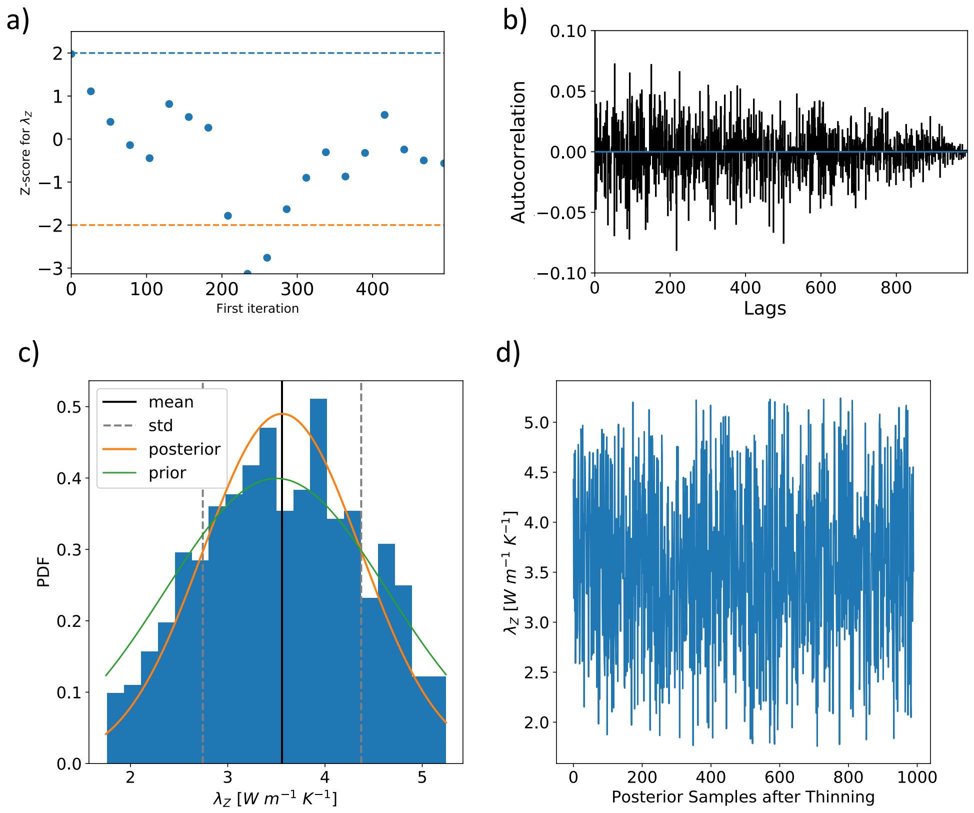

Fig. S7. Posterior Analysis of the Zechstein (Z). Shown are the a) Geweke Plot b) autocorrelation, c) posterior parameter distributions, and d) the trace.

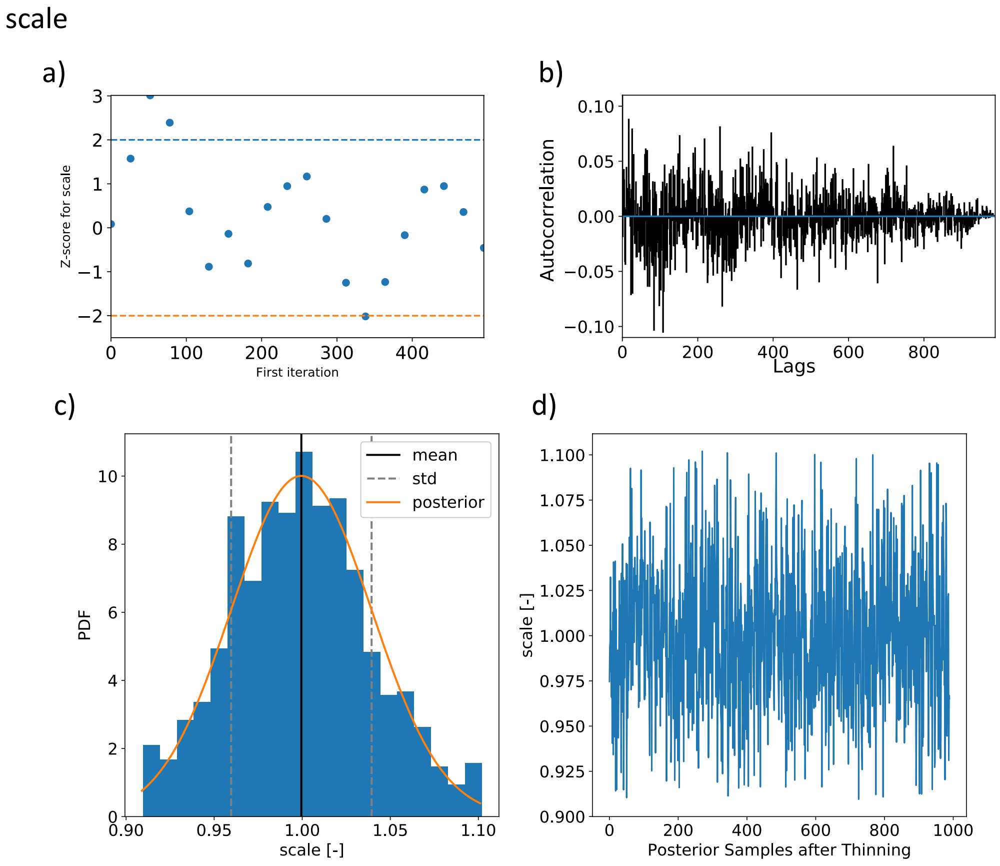

Fig. S8. Posterior Analysis of the scaling parameter for the lower boundary condition. Shown are the a) Geweke Plot b) autocorrelation, c) posterior parameter distributions, and d) the trace.

REFERENCES

1. V. Noack, M. Scheck-Wenderoth, and M. Cacace, "Sensitivity of 3D thermal models to the choice of boundary conditions and thermal properties: a case study for the area of Brandenburg (NE German Basin)," Environ. Earth Sci. **67**, 1695–1711 (2012).
2. V. Noack, M. Scheck-Wenderoth, M. Cacace, and M. Schneider, "Influence of fluid flow on the regional thermal field: results from 3D numerical modelling for the area of Brandenburg (North German Basin)," Environ. earth sciences **70**, 3523–3544 (2013).
